# Nitrogen and metal pollution in the southern Caspian Sea: a multiple approach to bioassessment

**DOI:** 10.1007/s11356-020-11243-8

**Published:** 2020-11-06

**Authors:** Maria Letizia Costantini, Homira Agah, Federico Fiorentino, Farnaz Irandoost, Francisco James Leon Trujillo, Giulio Careddu, Edoardo Calizza, Loreto Rossi

**Affiliations:** 1 Department of Environmental Biology, Sapienza University of Rome, Via dei Sardi 70, 00185 Rome, Italy; 2 National Inter-University Consortium for Marine Sciences (CoNISMa), Piazzale Flaminio 9, 00196 Rome, Italy; 3 Iranian National Institute for Oceanography and Atmospheric Sciences (INIOAS), No. 3, Etemadzadeh St., Fatemi Ave, Tehran, 1411813389 Iran; 4 University of Lima, Av. Javier Prado Este 4600, 15023 Santiago de Surco, Peru

**Keywords:** Coastal waters, Macroalgae, Sediment, Nitrogen stable isotopes, Metals, Bioassessment

## Abstract

The Caspian Sea hosts areas of high ecological value as well as industrial, leisure, and agricultural activities that dump into the water body different kinds of pollutants. In this complex context, a proper description of the origin and potential sources of pollution is necessary to address management and mitigation actions aimed at preserving the quality of the water resource and the integrity of the ecosystems. Here, we aimed at detecting sources of both nitrogen inputs, by N stable isotope analysis of macroalgae, and metals in macroalgae and sediments in two highly anthropized coastal stretches at the Iranian side of the Caspian Sea. Sampling was done near the mouth of rivers and canals draining agricultural and urbanized areas. In the westernmost waters, facing a port city, low macroalgal δ^15^N signatures indicated industrial fertilizers as the principal source of pollution. By contrast, in the central coastal waters, facing touristic areas, the high macroalgal δ^15^N indicated N inputs from wastewaters. Here the lowest dissolved oxygen concentrations in waters were associated with excess dissolved inorganic nitrogen. Metal concentrations varied largely in the study areas and were lower in macroalgae than in sediments. Localized peaks of Pb and Zn in sediments were observed in the central coastal sites as probable byproducts of mining activity transported downstream. By contrast, Cr and Ni concentrations were high in all sampling sites, thus potentially representing hazardous elements for marine biota. Overall, macroalgal δ^15^N coupled with metal analysis in macroalgae and sediments was useful for identifying the main sources of pollution in these highly anthropized coastal areas. This double approach in comprehensive monitoring programs could thus effectively inform stakeholders on major environmental threats, allowing targeted management measures.

## Introduction

Aquatic systems are often affected by anthropic activities taking place both on land and nearshore (Shahrban and Etemad-Shahidi 2010; Sohrabi et al. 2010), which poses a threat for the structure and functioning of coastal ecosystems (Halpern et al. 2008; Paerl et al. 2014). Indeed, industrial and agricultural activities, as well as wastewater discharges associated with resident population and tourism, dump different types of inputs, notably nutrients (Dailer et al. 2010; Halpern et al. 2008) and metals (Goher et al. 2014; Pekey et al. 2004), which can reduce the quality of water up to the inability to use it. In particular, the release of excess concentrations of nitrogen and phosphorus can result in coastal eutrophication (Howarth and Marino 2006; Paerl et al. 2014), and consequent algal blooms, hypoxia, and fish kills (Diaz and Rosenberg 2008; Paerl et al. 2016). In parallel, metals can directly and indirectly affect the aquatic biota (Adel et al. 2017) and, through biomagnification along food chains (Goher et al. 2014; Saghali et al. 2014), can reach humans with consequent risks for health (Agah et al. 2011; Dadar et al. 2016; Hosseini et al. 2013).

Among others, closed seas represent peculiar habitats of high ecological value, which are highly exposed to pollution impacts due to confinement and a long residence time of their water mass (Bastami et al. 2015; de Mora et al. 2004). Situated in the western Asia, the Caspian Sea is the largest closed water body on our planet. Due to its long isolation time, it shows a high level of endemism and offers a wide range of ecological niches (Bastami et al. 2015). On the other hand, being a land-locked system (Sohrabi et al. 2010), pollutants resulting from human activities persist in the water body, undermining the water quality and associated ecosystem services (de Mora et al. 2004). Various sources of pollution, such as river discharge, onshore industrial and municipal wastewater, and offshore and onshore oil extraction, threaten the Caspian Sea. Its water quality is also influenced by the water level fluctuations, which can increase water pollution from the flooded coastal zones, when water level rises, and can determine strong impacts on coastal settlements and agriculture. In recent decades, the water mass is reducing due to increasing evaporation rate (Chen et al. 2017), mainly affecting the shallow northern side, but having also adverse effects on the southern part of the water body where the majority of the water mass is dislocated (Chen et al. 2017). This sector hosts areas of high ecological value (Sadeghi et al. 2012). It also represents a crucial area for human activities, including tourism, fish farming, agriculture, manufacturing, and oil extraction (Abadi et al. 2018; Ebadi and Hisoriev 2017; Hasani et al. 2017), which are increasing coastal eutrophication (Irankhah et al. 2016; Sadeghi et al. 2013) and metal pollution (Bastami et al. 2015; de Mora et al. 2004; Hosseini et al. 2013; Zonn 2005). The associated increasing frequency of algal blooms (Makhlough et al. 2017; Nemati et al. 2017) and metal concentrations in fish (Dadar et al. 2016) is cause of environmental and public health concerns.

In this complex context, a proper description of potential pollution sources, origin, and dispersion is challenging but mandatory to address management and mitigation actions aimed at preserving the quality of the water resource and the integrity of the underlying ecological system. Indeed, while long-range transport of pollution may represent a major issue for the management of the water resource in wide and transnational water basins (as it is the case for the Caspian Sea), the identification of local pollution sources is necessary to achieve an effective management by the regional and national authorities. For this purpose, direct chemical analysis of pollutants in waters can have significant limitations. In particular, nutrients dilute rapidly in water and are absorbed by primary producers and sediments, thus limiting the spatial and temporal resolution of the water analysis. Moreover, the chemical analysis of N in waters does not allow to trace the source as it cannot discriminate against the “organic” (i.e., from wastewaters, manure) or “inorganic” origin (i.e., from industrial fertilizers) of the human-related N input (Gartner et al. 2002). This can potentially limit the efficiency of management actions for the pollution control. Compared to the atmospheric N_2_, the organic N sources show a greater ^15^N:^14^N ratio, whereas the inorganic N sources have a lower ^15^N:^14^N ratio (Orlandi et al. 2017; Sulzman 2007). This difference in N isotopic composition is translated in a different isotopic signature (δ^15^N) of the organic and inorganic N sources: δ^15^N ≥ 6‰ for wastewaters and manure (Dailer et al. 2010; Risk et al. 2009; Titlyanov et al. 2011) and − 3‰ ≤ δ^15^N ≤ + 3‰ for the industrial fertilizers (Dailer et al. 2010; Lapointe and Bedford 2007; Wang et al. 2016).

Nitrogen stable isotope signature of primary producers, especially macroalgae in brackish and marine ecosystems (Jona-Lasinio et al. 2015; Orlandi et al. 2014, 2017; Rossi et al. 2018; Vizzini et al. 2005) and epilithon in freshwater ecosystems (Bentivoglio et al. 2016; Fiorentino et al. 2017), has been recently recognized as a robust technique for the environmental monitoring of the organic and inorganic sources of the N inputs in waters (Dailer et al. 2010; Heaton 1986; Korom 1992; Kreitler and Jones 1975; Kreitler 1979; Mariotti et al. 1982; Rossi et al. 2018). Macroalgae directly assimilate N from the water, and their isotopic fractionation is small or null (Dailer et al. 2010; Orlandi et al. 2017). Thanks to these properties, the macroalgal δ^15^N signatures directly reflect the origin of the N inputs that entered in the water body, regardless of the N concentration in waters (Orlandi et al. 2017), and can indicate the principal anthropic source in heterogeneous landscapes (Calizza et al. 2020). This ability is crucial in some coastal areas of southern Caspian Sea where, given the proximity of various human activities, the management of pollution can be less effective without a robust determination of the N source.

Macroalgae are widely recognized also as a powerful tool for the monitoring of metal pollution in marine ecosystems due to their ability to bind metals (Astorga-España et al. 2008), which can lead the concentrations of metals to several orders of magnitude higher than in waters (Villares et al. 2001). In addition, the concentrations of metals in macroalgae are less variable than in waters, where they are influenced by hydrological factors (Billah et al. 2017). Thus, a reliable representation of metal pollution through water metal concentration analysis will require a greater number of samplings and samples, with consequent increasing costs and time-consuming procedures (Villares et al. 2001). Macroalgae are common in polluted sites and easy to sample (Astorga-España et al. 2008; Dailer et al. 2010). Their use is thus successful in order to obtain spatial and temporal information on bioavailability of metal pollutants (Chakraborty et al. 2014). However, the complete description of pollution pathways in coastal areas needs to take into account also pollutants’ concentration in the sediments (Bastami et al. 2017). Indeed, sediments are sink for metals released into an environment and provide a stable record of deposition history (Agah et al. 2012; Bastami et al. 2014; Goher et al. 2014; Villares et al. 2001). In addition, since benthic, epibenthic, and infaunal biota can accumulate metals from sediments, the sediment compartment, together with macroalgae, can represent a potential “gateway” for metals to higher trophic levels (Mendoza-Carranza et al. 2016).

Aim of the present study is to use the stable isotope analysis of macroalgae and analysis of metals in macroalgae and surface sediments to detect the main pollution sources affecting the heavily anthropized coastal area of the southern Caspian Sea, a unique ecosystem threatened by multiple anthropogenic stressors. Despite the large use of the algal δ^15^N approach in the environmental bioassessment of aquatic ecosystems, to our knowledge this approach has never been used to identify the main sources of N inputs in the Iranian Caspian Sea, where the multitude of human activities affecting coastal areas (e.g., agriculture, tourism, and fish farming) makes other analytical approaches poorly effective. We hypothesize that wastewaters and fertilizer N inputs are reflected by the macroalgal δ^15^N values (Jona-Lasinio et al. 2015; Orlandi et al. 2014; Rossi et al. 2018), which can thus indicate the main sources of this nutrient in the Caspian coastal waters. This in turn can provide useful information to the stakeholders for subsequent management actions. In parallel, we measured metal concentrations in macroalgae and sediments in order to (i) compare pollution levels between these two compartments supporting the coastal food web and (ii) provide a comprehensive description of anthropogenic pollution affecting the southern coastal area of the Caspian Sea.

## Materials and methods

### Study area

The Caspian Sea, denoted as a sea or a lake, is the largest inland water body in the world, with a total surface of ≈ 371,000 km^2^ and a maximum depth of 1025 m. It accounts for ≈ 40–44% of the total lacustrine waters in the world, and it is surrounded by Azerbaijan, Federation of Russia, Islamic Republic of Iran, Kazakhstan, and Turkmenistan.

**Fig. 1:**
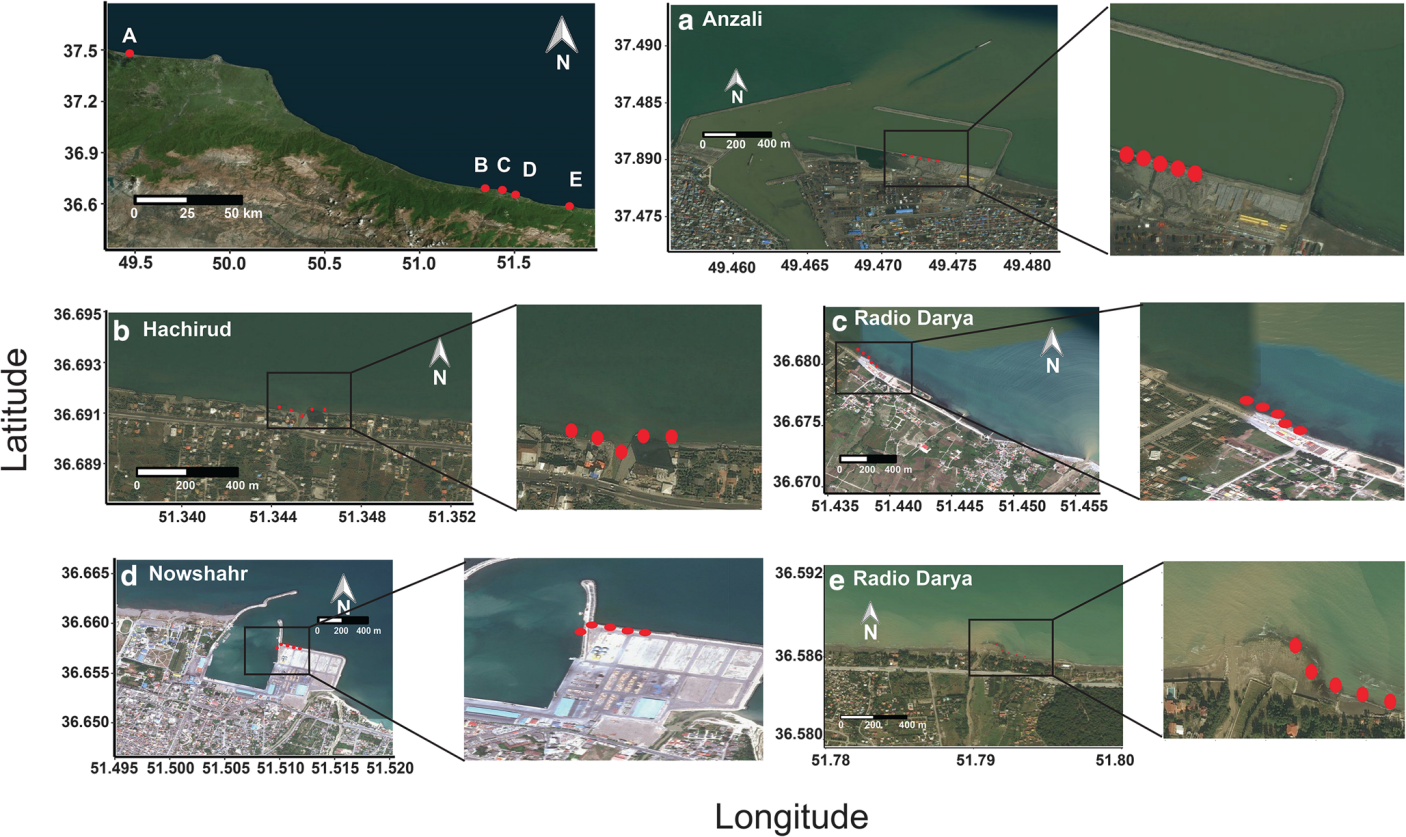
Map of the study area. General view of the Iranian coast of the Caspian Sea, including the Gilan and Mazandaran Provinces. Each letter corresponds to a sampling site: (**A**) Anzali, (**B**) Hachirud, (**C**) Radio Darya, (**D**) Nowshahr, (**E**) Sisangan. The red points correspond to the sampling stations in each sampling site and are placed 50 m apart from each other along a transect

We focused on the coastal waters of two densely populated areas of the southern Caspian Sea (Iran). Sampling was planned in order to intercept anthropic inputs of nitrogen and metal pollutants by rivers and canals draining agricultural and urbanized areas. Five sampling sites were selected near the mouth or within the plume of waterways, where macroalgae were present (Fig. 1). The sampling sites included the city of Bandar-e Anzalī (37.4807° N and 49.4715° E), in the Gilan Province, and the cities of Hachirud (36.6911° N and 51.3454° E), Radio Darya (36.6816° N and 51.4364° E), and Nowshahr (36.6540° N and 51.5061° E) and the Sisangan beach (36.5862° N and 51.7903° E) in the Mazandaran Province. The sampling site at Bandar-e Anzalī (hereafter Anzali) is located in the sandy shoreline of the city (around 150,000 inhabitants), and it is potentially exposed to wastewater and agricultural inputs, as well as pollution derived by the harbor and local industry (Sadeghi et al. 2012, 2013). This area is affected by the discharge of the Sefid-Rud River (the “White River”), which is the second river in Iran, 670 km long and with a drainage basin of 13,450 km^2^ and dams. Along its lowland stretch (around 55 km in length), the river crosses a highly cultivated area, where crops cover the majority of the drainage basin and reach the river banks (Nemati et al. 2017). The sampling site of Anzali is placed 0.71 km apart the river mouth. Hachirud, Radio Darya, and Nowshahr are located in the urbanized area of Chalus (around 70,000 inhabitants). In this area, urban aggregates are surrounded by agricultural activities, especially tea and rice fields, which are more abundant at Hachirud than at Radio Darya and Nowshahr that are popular touristic destinations (Irankhah et al. 2016). The sampling site of Hachirud directly faces the channel mouth. Due to the wind-driven sea currents, the port Nowshahr is affected by the supply of sediments (≈ 40,000 m^3^ per year) from the Mashalak River (Mahmoodi et al. 2020), which is 0.74 km distant from Nowshahr sampling site. The area of Chalus is affected by the discharge of Chalus River, which is 92 km long and has a drainage basin of 1710 km^2^ flowing into the Caspian Sea near Radio Darya after crossing the city of Chalus. The Radio Darya sampling site is located 1.70 km apart the river mouth. Along its upstream stretch the Chalus River crosses an industrial area. Here, the Sorb Dona is dedicated to mineral extraction and represents a potential source of metal pollution affecting water quality (Jelodar et al. 2012; Amini Rad et al. 2013). The Sisangan sampling site is located close to the Sisangan National forest in the Sisangan National Park, established in 1965, where human activity is regulated. Here the Kojur River flows in the Caspian Sea after crossing a deciduous forest (Sadeghi and Kheirfam 2015). The Sisangan sampling site, 0.24 km from the river mouth, was expected to be the least affected by direct anthropogenic inputs of all sampling sites.

### Field sampling procedures

Samplings were carried out between May and June 2016. At each of the five sampling sites (Anzali, Hachirud, Radio Darya, Nowshahr, and Sisangan), five sampling stations, 50 m apart from each other, were selected along a transect (Fig. 1). At each sampling station, samples of algae and sediments were collected. Specifically, at each station, three samples of *Enteromorpha* spp*.* were randomly collected by hand, between 0.5 and 1 m depth, and used for the isotopic comparison across the five sampling sites and for metal analysis. Occasionally *Spirogyra* spp*.* and *Sargassum* spp*.*, which can represent a food source for primary consumers, were also found and analyzed for metal concentrations. After collection, algal samples were placed in clean plastic bags, labeled, and carried to laboratory in ice boxes. Then, samples were washed up to remove mud, clay, and salt, and air-dried. For the analysis of metals, in each station three samples of surface sediments (0–5 cm) were collected by using an Ekman grab (model 437 200, Hydro Bios). After collection, sediment samples were stored in clean polyethylene plastic bottles, labeled, and carried to the laboratory in ice boxes for further treatment. The sediment samples were lyophilized, sieved, and fractions smaller than 63 μm were transferred in clean dark glass bottles and kept frozen (at − 20 °C) prior to chemical analyses (Wolf-Welling et al. 2002). Water samples, three for each sampling station, were also collected for nitrogen and phosphorus analysis. During sampling, pH, dissolved oxygen (DO, mg/l), and temperature (°C) in water were recorded by using portable field probes.

### Laboratory procedures

Dissolved inorganic nitrogen (DIN, mg/l) as $\mathrm{N}{\mathrm{H}}_4^{+}$ (phenate method, MOOPAM 1999) + $\mathrm{N}{\mathrm{O}}_2^{-}$ (colorimetric method, MOOPAM 1999) + $\mathrm{N}{\mathrm{O}}_3^{-}$(cadmium reduction method, MOOPAM 1999) and dissolved inorganic phosphorus (DIP, mg/l) as ${\mathrm{PO}}_4^{3-}$ (vanadomolybdophosphoric acid colorimetric method, MOOPAM 1999) were determined by a spectrophotometer (DR-2500 Model HACH, USA). Samples of macroalgae were processed and analyzed for stable isotope analysis in the Laboratory of Trophic Ecology, Dept. of Environmental Biology, Sapienza University of Rome (Italy). Samples were conserved at 60 °C in an oven for 72 h and then ground to a fine homogenous powder using a ball mill (Fritsch Mini-Mill Pulverisette 23 with a zirconium oxide ball). For each sample, two sub-replicates (2.0 ± 0.2 mg) were weighed, pressed into ultra-pure tin capsules (Fiorentino et al. 2017; Orlandi et al. 2014; Rossi et al. 2018), and analyzed using an Elementar Vario Micro-Cube elemental analyzer (Elementar Analysensysteme GmbH, Germany) coupled with an IsoPrime100 isotope mass ratio spectrometer (Isoprime Ltd., Cheadle Hulme, UK). The nitrogen stable isotope ratio (^15^N:^14^N) was expressed in *δ* units, i.e., parts per thousand deviations from international standards (atmospheric N_2_), in accordance with Ponsard and Arditi (2000) equation (Eq. 1):

1 $$ \delta R\left({\mbox{\fontencoding{U}\fontfamily{wasy}\selectfont\char104}} \right)=\left[\frac{\left({R}_{\mathrm{sample}}-{R}_{\mathrm{standard}}\right)}{R_{\mathrm{standard}}}\right]\ast {10}^3 $$

where *R* is the heavy-to-light isotope ratio of the element. The internal laboratory standard was IAEA-600 Caffeine. Measurement errors were found to be typically smaller than ± 0.5‰.

In accordance with Fiorentino et al. (2017), we derived four impact classes based on the macroalgal δ^15^N values, indicative of different N inputs: “inorganic input” (δ^15^N < 3‰), “non-impacted” (3‰ ≤ δ^15^N ≤ 6‰), “moderate organic input” (6‰ < δ^15^N ≤ 9‰), and “high organic input” (δ^15^N > 9‰).

The analysis of metals in sediment and macroalgae were performed by the Institute for Nano Science and Nanotechnology at Sharif University of Technology (Iran). In this research, we considered metals with well-known anthropic origins and metals that are naturally found in the Iranian Caspian Sea sediments (for further details, see Agah et al. 2011) and can limit the algal growth and blooms (e.g., Fe; Bruland et al. 2001; Facey et al. 2019; Sunda 2006). High concentrations of these metals in sediments and macroalgae can indicate metal pollution due to anthropic activities (Caccia et al. 2003; Caliceti et al. 2002; Chakraborty et al. 2014; Ghosh et al. 2019), and by biomagnification, they can become potentially toxic (Adel et al. 2017; Dadar et al. 2016).

Samples of macroalgae for metal analysis were grinded using a porcelain mortar and kept frozen until analysis. One-gram dry sample was digested by 10 ml HNO_3_ and 1 ml H_2_O_2_ (30% Merck, Suprapur) for 2 h at 90 °C. The digested samples were cooled at laboratory temperature, filtered through a Whatman filter paper (No. 42) and diluted to 50 ml with distilled deionized water. Metal concentrations were determined by ICP-OES (series No ICAP6000, Spectro Arcos, Ametek, Termo Company, England). For each element, the Biosediment Accumulation Factor (BSAF), i.e., the bioavailability of the element, was evaluated according to the following equation (Eq. 2, Alahverdi and Savabieasfahani 2012):

2 $$ \mathrm{BSAF}=\frac{X_{\mathrm{macroalgae}}}{X_{\mathrm{sediment}}} $$

where *X* is the concentration of a given element.

The grain size of sediments was measured using laser (laser scattering particle size distributer analyzer LS-950 Model by Horiba) and shaker (FRITSCH Analysette 3 PRO) instruments for silty-muddy and sandy sediments. Of the sediment sample, 0.5 g was weighed in a Teflon vessel and digested using HNO_3_ (65% Merck Suprapur) and HCl (1:3 v/v) at 85 °C for 3 h (Al-Mur et al. 2017). The acidified samples were cooled for 1 h at laboratory temperature, filtered through a Whatman filter paper (No. 42), and diluted to 50 ml with distilled deionized water. For each digestion program, a “blank” was also prepared.

Metals (Al, Ba, Ca, Co, Cr, Cu, Fe, K, Mg, Mn, Na, Ni, Pb, Ti, V, and Zn), as well as some other elements (P and S), were analyzed using inductively coupled plasma optical emission spectrometry ICP-OES after acid digestion. Quantification of elements was based upon calibration curves obtained from different standard solutions prepared at 2, 20, 200, 500, 1000, and 2000 μg/l (Merck, code No. 1.11355.0100).

For Cr, Cu, Ni, Pb, and Zn, it was possible to compare the observed concentrations with values proposed by the sediment Standard Quality Guidelines (SQG, Perin et al. 1997), also utilized by Pekey et al. (2004, Marmara Sea) and Agah et al. (2011, Caspian Sea). This classification provides threshold values for three different classes of impact: non-polluted (NP), moderately polluted (MP), and heavily polluted (HP). Observed concentrations were also compared with the “threshold effect level.” The TEL provides threshold values that can be related with potential toxic effects of each metal on the marine biota (Long et al. 1998).

### Statistical analysis

To test the occurrence of differences in macroalgal δ^15^N values and DIP concentrations in water among sampling sites, we performed one-way ANOVA. To deal with small differences in sampling size, the ANOVA was estimated as a linear regression model (Quinn and Keough 2002). In this approach, the macroalgal δ^15^N values and DIP concentrations were in function of the qualitative variable “Site” (dummy variable with five levels, James et al. 2013). If differences among sampling sites emerged from the ANOVA table, a Tukey’s honest significant difference test was used. ANOVA assumptions on model residuals normality and homoscedasticity were assessed respectively with the Shapiro-Wilks test and the Bartlett’s test. No data transformations were applied to the data to achieve model residuals normality and homoscedasticity. Principal component analysis was performed to reduce the number of variables by associating elements in surface sediments and thus determining common features. Then Pearson’s linear correlations between element concentrations in sediments were estimated. In order to explore potential coupling between compartments, we estimated the Pearson’s linear correlations between element concentrations in sediments, and in macroalgae. We also calculated Pearson’s linear correlation between δ^15^N values and element concentrations in algae to test common sources of N and metal pollution. For all the linear correlations, we tested that the correlation coefficients were not zero (cor.test function in R). In order to explore the relationships between the origin and concentrations of nitrogen and the effects on the oxygen concentration in waters, we estimated linear regressions between macroalgal δ^15^N and dissolved inorganic nitrogen (DIN) and between DIN and dissolved oxygen (DO). The normal distributions of the linear regression’s residuals were assessed with the Shapiro-Wilks test, and homoscedasticity was graphically checked.

We tested the existence of a linear correlation between DO and water temperature (cor.test function in R). For all the models and tests, the confidence level was set at *α* = 0.05. Data analysis was performed with the open-source software R 3.4.2 (R Core Team 2017) and ade4 package (Dray and Dufour 2007).

## Results

### δ^15^N in macroalgae and physico-chemical parameters in water

**Fig. 2:**
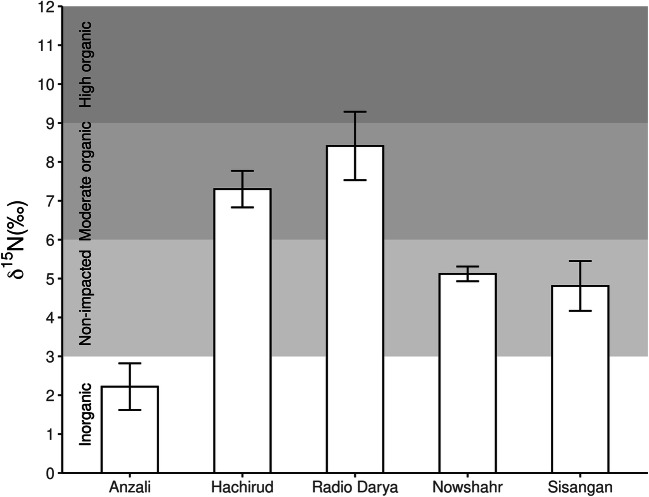
Bar plots of mean (± standard error) δ^15^N values in macroalgae at different sampling sites. Isotopic thresholds denoting different sources and levels of anthropogenic N pollution are indicated in the background of the image. “Inorganic” pollution, δ^15^N < 3‰; “non-impacted”, 3‰ ≤ δ^15^N ≤ 6‰; “moderate organic” pollution, 6‰ < δ^15^N ≤ 9‰; “high organic” pollution, δ^15^N > 9‰

δ^15^N values of macroalgae varied across sampling sites, thus detecting different origins of the N inputs (Fig. 2). In the western coastal area (Anzali), nitrogen signatures fell in the inorganic range, with a mean (±SE) δ^15^N value of 2.22‰ (± 0.60). In the central area, δ^15^N values reached the highest values falling in the “moderate organic” and “high organic” ranges near Hachirud and Radio Darya, with an average of 7.30‰ (± 0.47) and 8.41‰ (± 0.88), respectively (Fig. 2). East of this area, nitrogen signatures fell in the “non-impacted” range with an average of 5.12‰ (± 0.19) and 4.81‰ (± 0.64) near Nowshahr and Sisangan, respectively. ANOVA highlighted significant differences in the macroalgal δ^15^N signatures among sampled areas (*F* value = 15.76, *p* < 0.05) with ≈ 70% of deviance explained. The Shapiro-Wilks test and Bartlett’s test confirmed ANOVA residuals normality and homoscedasticity (*p* values > 0.05). Values found in the “inorganic impacted” site (Anzali) were statistically different from those of the other sites (Tukey’s pairwise comparisons, *p* < 0.05). Furthermore, values in the two non-impacted sites were similar (Nowshahr and Sisangan, *p* > 0.05) and significantly different from those of “organic impacted” sites (Tukey’s pairwise comparisons, both *p* < 0.05).

**Fig. 3:**
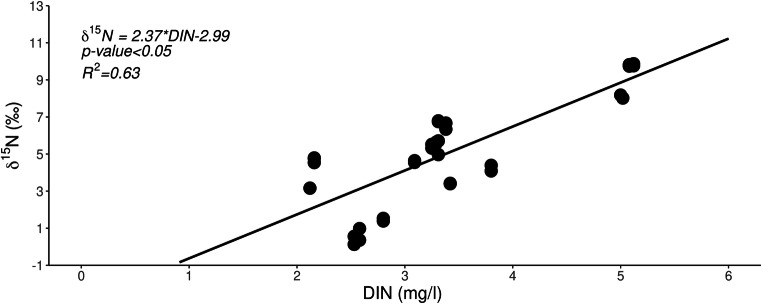
Linear regressions between δ^15^N (‰) values in macroalgae against dissolved inorganic nitrogen (DIN, mg/l). The linear regression highlights that DIN increased in the touristic areas, and its origin was related with wastewaters

δ^15^N values were positively related with DIN (Fig. 3), while DO and DIN were negatively related (DO = − 2.96*DIN + 17.13, *p* value < 0.05, *R*^2^ = 0.89). DO did not vary with water temperature (Pearson’s coefficient, *p* > 0.05), which was rather constant between sampling sites (Table 1). Sites affected by anthropogenic N inputs of both “inorganic” and “organic” origin showed higher DIP concentrations than the two non-impacted sites (ANOVA *p* < 0.05; Tukey’s pairwise comparisons *p* < 0.05, Shapiro-Wilks and Bartlett’s test, *p* > 0.05).

**Table 1:** Water parameters (mean ± standard error) at various sampling sites in the southern Caspian Sea

Site	DO (mg/l)	DIN (mg/l)	DIP (mg/l)	pH	T (°C)
Anzali	11 ± 0.31	2.4 ± 0.08	0.7 ± 0.18	7.7 ± 0.04	25 ± 0.04
Hachirud	4.7 ± 0.96	4.2 ± 0.47	0.7 ± 0.34	8.3 ± 0.01	25 ± 0.12
Radio Darya	4 ± 0.96	4.5 ± 0.38	1 ± 0.27	8.2 ± 0.14	25 ± 0.10
Nowshahr	7 ± 0.20	3 ± 0.04	0.05 ± 0.01	7.7 ± 0.02	25 ± 0.04
Sisangan	6 ± 0.11	3.5 ± 0.09	0.05 ± 0.01	7.8 ± 0.01	25 ± 0.02

DIN and DIP indicate the concentration of dissolved nitrogen and phosphorus, respectively

*DO* dissolved Oxygen, *DIN* dissolved inorganic nitrogen, *DIP* dissolved inorganic phosphorus, *T* temperature

### Metal concentrations in sediments

Sandy-silty sediments characterized all sampling stations. Overall, metals had very different mean concentrations and ranked with the following decreasing pattern: Fe (72,000 mg/kg) > Ca (69,000 mg/kg) > Al (30,000 mg/kg) > Mg (22,000 mg/kg) > Ti (9000 mg/kg) > K (7000 mg/kg) > Na (6500 mg/kg)> Mn (2200 mg/kg) > V (500 mg/kg) > Cr (400 mg/kg) > Ba (200 mg/kg) > Zn (140 mg/kg) > Ni (60 mg/kg) > Pb (40 mg/kg) > Co (30 mg/kg) > Cu (20 mg/kg). The mean concentration of each element varied across sampling sites (Table 2).

**Table 2:** Average ± standard error of element concentrations in sediments at the five sampling sites in the southern Caspian Sea

Element	Anzali (A)	Hachirud (H)	Radio Darya (R)	Nowshahr (N)	Sisangan (S)	Maximum
Al (I)	45,000 ± 2200	15,600 ± 550	11,300 ± 490	43,000 ± 240	37,300 ± 1600	A
Ca (I)	99,000 ± 2800	44,000 ± 2600	26,000 ± 880	90,000 ± 4200	84,000 ± 2400	A
Fe (II)	63,000 ± 2100	91,000 ± 5700	98,000 ± 4400	40,000 ± 2500	67,000 ± 3300	R
Mg (I)	30,000 ± 1800	16,800 ± 680	9200 ± 470	26,000 ± 1400	29,000 ± 1700	A
Mn (II)	2200 ± 150	3500 ± 200	3000 ± 170	800 ± 50	1500 ± 80	H
Na (I)	10,000 ± 370	2500 ± 70	1500 ± 50	10,000 ± 260	8700 ± 310	A
Ti (I)	15,700 ± 780	3600 ± 100	6300 ± 340	14,000 ± 640	7000 ± 240	A
K (I)	10,400 ± 320	2000 ± 100	1500 ± 70	12,000 ± 440	9000 ± 440	N
S (III)	730 ± 27	460 ± 8	400 ± 16	420 ± 23	450 ± 22	A
P (III)	750 ± 30	810 ± 39	700 ± 39	630 ± 33	640 ± 30	H
Ba (I)	240 ± 12	90 ± 4	120 ± 5	270 ± 9	210 ± 10	N
Cr (II)	300 ± 14	700 ± 35	700 ± 23	170 ± 6	300 ± 16	H
V (II)	260 ± 12	800 ± 39	900 ± 28	95 ± 4	270 ± 9	R
Ni (II)	51 ± 2	80 ± 3	80 ± 7	40 ± 1	50 ± 2	H
Pb (II)	30 ± 1	61 ± 2	62 ± 3	30 ± 1	30 ± 1	R
Zn (II)	80 ± 3	250 ± 8	200 ± 6	45 ± 2	100 ± 6	H
Co (II)	25 ± 1	45 ± 2	40 ± 1	15 ± 1	20 ± 1	H
Cu (II)	20 ± 1	45 ± 2	30 ± 1	10 ± 1	15 ± 1	H

Element concentrations are expressed in mg/kg. “Maximum” in the last column indicates the site (first letter of site name, as indicated in brackets) where the highest concentration of each element was recorded

I, II, and III associated to each element indicate different groups in which metals were grouped according to the PCA analysis. *A* Anzali, *H* Hachirud, *R* Radio Darya, *N* Nowshahr, *S* Sisangan

**Fig. 4:**
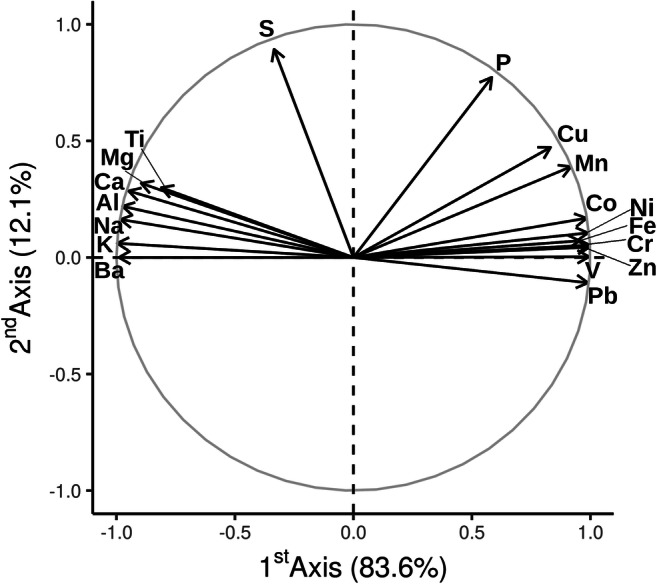
Circle plot where each element is represented as a vector. The coordinates of the arrowhead are the Pearson’s correlation coefficients between the elements and the two principal components. The closer a vector to an axis, the higher the contribution to the principal component. Percentages of variance explained by each component are reported. On the first axis, two distinct groups cluster in opposite directions, suggesting different origins of the elements

The principal component analysis grouped elements in three groups (Fig. 4). The first two groups included metals and were separated along the first PCA axis (≈ 84% of total variance explained), whereas the non-metal elements (P and S, group III) mainly scattered on the second PCA axis (≈ 12% of total variance). Notably, group II included metals of anthropic origin that are expected to affect the marine biota depending on concentration. Positive Pearson’s linear correlation coefficients were found among elements within each group, while negative coefficients were found between the first two groups (Table 3).

**Table 3:** Pearson’s linear correlation coefficients (*r*) between elements

	Al (I)	Ba (I)	Ca (I)	K (I)	Mg (I)	Na (I)	Ti (I)	Co (II)	Cr (II)	Cu (II)	Fe (II)	Mn (II)	Ni (II)	Pb (II)	V (II)	Zn (II)	P (III)	S (III)
Al (I)	–	0.96*	0.99*	0.99*	0.96*	0.99*	0.83	− 0.92*	− 0.96*	− 0.70	− 0.91*	− 0.81	− 0.94*	− 0.99*	− 0.97*	− 0.96*	− 0.40	0.52
Ba (I)		–	0.92*	0.98*	0.84	0.96*	0.87	− 0.97*	− 0.98*	− 0.85	− 0.94*	− 0.90*	− 0.96*	− 0.97*	− 0.97*	− 0.99*	− 0.60	0.36
Ca (I)			–	0.97*	0.98*	0.99*	0.78	− 0.89*	− 0.94*	− 0.63	− 0.89*	− 0.76	− 0.92*	− 0.98*	− 0.95*	− 0.92*	0.55	− 0.18
K (I)				–	0.92*	0.99*	0.82	− 0.97*	− 0.99*	− 0.78	− 0.96*	− 0.89*	− 0.98*	− 0.99*	− 0.99*	− 0.99*	− 0.52	0.37
Mg (I)					–	0.96*	0.66	− 0.84	− 0.88*	− 0.56	− 0.81	− 0.70	− 0.88*	− 0.94*	− 0.91*	− 0.86	− 0.26	0.55
Na (I)						–	0.80	− 0.94*	− 0.97*	− 0.73	− 0.92*	− 0.84	− 0.96*	− 0.99*	− 0.98*	− 0.97*	− 0.44	0.47
Ti (I)							–	− 0.73	− 0.78	− 0.59	− 0.77	− 0.62	− 0.72	− 0.80	− 0.76	− 0.83	− 0.28	0.61
Co (II)								–	0.99*	0.88*	0.96*	0.97*	0.99*	0.95*	0.98*	0.98*	0.69	− 0.16
Cr (II)									–	0.84	0.97*	0.94*	0.99*	0.98*	0.99*	0.99*	0.61	− 0.27
Cu (II)										–	0.76	0.93*	0.85	0.78	0.80	0.87	0.92*	0.06
Fe (II)											–	0.92*	0.96*	0.92*	0.97*	0.95*	0.55	− 0.18
Mn (II)												–	0.95*	0.86	0.92*	0.93*	0.82	0.06
Ni (II)													–	0.97*	0.99*	0.98*	0.64	− 0.21
Pb (II)														–	0.98*	0.98*	0.50	− 0.44
V (II)															–	0.98*	0.56	− 0.30
Zn (II)																–	0.63	− 0.31
P (III)																	–	0.42
S (III)																		–

Positive linear correlation coefficients between elements suggest common origins (Bastami et al. 2014, 2017)

^*^p < 0.05. I, II, and III indicate the different groups identified by PCA

According to the SQG (Table 4), high levels of Cr and Ni were present in all sites and TEL was exceeded. In the “inorganic impacted” area, concentrations of Cu, Pb, and Zn were below the level of pollution, whereas in the “organic impacted” sites (Hachirud and Radio Darya) high levels of Pb and Zn were found, exceeding the corresponding TEL, and Cu was in the moderate pollution class. Cu exceeded the TEL both in the “organic” and “inorganic impacted” sites. Although in Sisangan metals did not reach concentrations as high as those observed in the other sites, values fell in the high pollution range for Cr, and in the moderate pollution range for Ni and Zn. Here, Zn concentration was below the TEL.

**Table 4:** Average ± standard error of metal concentrations (in mg/kg) for metals included in the Sediment Quality Guidelines (SQG)

Sediment SQG classification				
Element	Site	mg/kg	Classification	Class
Cr	Anzali	300 ± 14	Cr < 25 non-polluted	HP
(TEL: 52.3)	Hachirud	700 ± 35	25 ≤ Cr ≤ 75 Moderately polluted	HP
	Radio Darya	700 ± 23		HP
	Nowshahr	170 ± 6	Cr > 75 heavily polluted	HP
	Sisangan	300 ± 16		HP
Cu	Anzali	20 ± 1	Cu < 25 non-polluted	NP
(TEL: 18.7)	Hachirud	45 ± 2	25 ≤ Cu ≤ 50 Moderate polluted	MP
	Radio Darya	30 ± 1		MP
	Nowshahr	10 ± 1	Cu > 50 heavily polluted	NP
	Sisangan	15 ± 1		NP
Ni	Anzali	51 ± 2	Ni < 20 Non-polluted	HP
(TEL:15.9)	Hachirud	80 ± 3	20 ≤ Ni ≤ 50 Moderate polluted	HP
	Radio Darya	80 ± 7		HP
	Nowshahr	40 ± 1	Ni > 50 heavily polluted	MP
	Sisangan	50 ± 2		MP
Pb	Anzali	30 ± 1	Pb < 40 non-polluted	NP
(TEL:30.2)	Hachirud	61 ± 2	40^.^ ≤ Pb ≤ 60 Moderate polluted	HP
	Radio Darya	62 ± 3		HP
	Nowshahr	30 ± 1	Pb > 60 heavily polluted	NP
	Sisangan	30 ± 1		NP
Zn	Anzali	80 ± 3	Zn < 90 non-polluted	NP
(TEL: 124)	Hachirud	250 ± 8	90 ≤ Zn ≤ 200 Moderate polluted	HP
	Radio Darya	200 ± 6		HP
	Nowshahr	45 ± 2	Zn > 200 heavily polluted	NP
	Sisangan	100 ± 6		MP

*TEL* threshold effect level (mg/kg), *NP* non polluted, *MP* moderately polluted, *HP* heavily polluted

### Element concentrations in macroalgae

On average, element concentrations in macroalgae (Table 5) decreased according to the following order: Ca (21,000 mg/kg) > Mg (2500 mg/kg) > Fe (1800 mg/kg) = Al (1800 mg/kg) > P (500 mg/kg) > K (430 mg/kg) > Mn (75 mg/kg) > Zn (60 mg/kg) > Ti (40 mg/kg) > Cu (30 mg/kg) > Cr (3 mg/kg) = V (3 mg/kg) = Ni (3 mg/kg) > Co (0.6 mg/kg).

**Table 5:** Descriptive statistics of element concentrations measured in macroalgae along the Iranian coast of the Caspian Sea

	Ca	Mg	Fe	Al	P	K	Mn	Zn	Ti	Cu	Cr	V	Ni	Co
Minimum	15,000	1500	760	890	350	220	30	30	20	10	2	0.01	1	0.01
1st quartile	18,000	2200	1100	1200	390	300	50	40	25	15	2	2	2	0.1
Median	21,000	2500	1900	1700	440	350	80	50	30	20	2	3	2	0.5
Mean	21,000	2500	1800	1800	500	430	75	60	40	30	3	3	3	0.6
3rd quartile	24,000	2700	2200	2200	600	540	100	70	30	20	3	3	3	1
Maximum	27,000 **a**	4000 **a**	3300 **b**	2700 **b**	940 **b**	860 **b**	110 **b**	100 **b**	150 **b**	80 **a**	12 **b**	6 **b**	5 **b**	2 **a**

Element concentrations are in mg/kg

Letters in bold below maximum values indicate the algal species where the maximum was observed. Samples belonging to the genus *Spirogyra* were excluded because they had the lowest metal concentrations. a, *Sargassum*; b, *Enteromorpha*

Concentrations of elements differed between macroalgal species (Table 5). Specifically, *Enteromorpha* showed the highest concentration for Fe, Al, P, Ti, Cr, V, and Ni (maximum values observed in Anzali), while Cr and Ni, which were abundant in the sediments, were present in low concentrations in all the other macroalgal species. The BSAF values were generally lower than 1, implying that the concentration observed in algae was lower than that observed in sediments for the corresponding element. Pearson’s linear correlation coefficients did not differ from zero between a specific element in macroalgae and the same element in sediments nor did they differ between δ^15^N and metal concentrations in macroalgae (*p* > 0.05).

The high Fe concentrations in *Enteromorpha* were found at all sampling sites, and high Cu levels were found close to the Sisangan National Park.

## Discussions

The Iranian side of the Caspian Sea is known to suffer from cultural eutrophication (Makhlough et al. 2017; Nemati et al. 2017) and metal pollution (Bastami et al. 2015; de Mora et al. 2004) due to multiple and often co-occurring inputs (Mehdinia et al. 2020; Nemati et al. 2017). Pollutants can derive from various human activities carried out both at sea and on land, which might impair water resources and human health, thus urging effective management actions. Our results obtained from stable isotope and metal analyses detected considerable spatial variation of pollution in the study area. Integration of the two approaches was crucial for depicting more fully the anthropic impact complexity and determining the main pollution sources. On one hand, the δ^15^N analysis of macroalgae allowed the classification of the coastal waters on the basis of the predominant origin of anthropogenic N input. On the other hand, analysis of sediments revealed potentially hazardous elements for marine biota and indicated distinct and spatially variable inputs of natural and anthropogenic metals into the sea.

Specifically, as determined in other coastal-marine ecosystems (e.g., Dailer et al. 2010; Rossi et al. 2018), the low δ^15^N values of the macroalgae (δ^15^N < 3‰) in the western marine coastal waters are indicative of anthropogenic inorganic N sources. The low values identify the agricultural-derivation inorganic fertilizers as the main source of N inputs in the Anzali area. Indeed agriculture (especially rice cultivation) together with other human activities (industrial and urban) has been invoked as causes of eutrophication in the Anzali wetlands (Sadeghi et al. 2012, 2013, and literature cited therein). Our results support the hypothesis of an agricultural origin of nitrates also in the Anzali tap water. This hypothesis was advanced by Ziarati et al. (2014) who, however, stated in their work that the identification of the origin of nitrates is a challenge as multiple sources may be involved.

The high δ^15^N values (δ^15^N > 6‰) of macroalgae found in the central sites (Hachirud and Radio Darya) can be mainly attributed to tourism and municipal wastewaters according to Dailer et al. (2010) and Rossi et al. (2018). These high values, which were associated with high dissolved nitrogen levels in seawaters, indicated respectively a moderate and a high organic origin of the N inputs suggesting the need of wastewater collection and treatment systems. East of this area, δ^15^N values indicated “non-impacted” conditions, according to current literature classification (Dailer et al. 2010; Fiorentino et al. 2017; Lapointe and Bedford 2007; Risk et al. 2009; Titlyanov et al. 2011; Wang et al. 2016). In particular, the values found in Sisangan are consistent with the location of the site near the Sisangan National Park, where human activities are strongly regulated. The increasing DIN concentration in the southern Caspian Sea coastal waters was related with increasing δ^15^N values of macroalgae indicating the organic origin of the N inputs. In such conditions, DO decreased (even close to the hypoxic threshold of 2 mg/l, Jessen et al. 2015), plausibly due to heterotrophic microbial respiration that is expected to increase with organic matter degradation (Diaz and Rosenberg 2008).

As regards metals in sediments, concentrations of Co and Cu were rather similar to relatively recent studies in the area (Karbassi et al. 2008; Sohrabi et al. 2010), whereas higher concentrations of Ni, Pb, and Zn were found in comparison with Sohrabi et al.’s finding (2010). According to the SQG classification, most of the sampling sites fall in the “moderately impacted” and “highly impacted” classes for various metals, indicating the influence of industrial, agriculture, and urban wastes in the study area (Karbassi et al. 2008; Sohrabi et al. 2010; Vesali Naseh et al. 2012). As observed for N inputs, the measured variability of most metal concentrations across sites suggests spatial differences in anthropogenic pollution affecting the southern Caspian Sea and provides site-specific baseline values of current conditions for future comparisons.

Overall, the PCA results highlighted that metals clustered in two groups: one (group I) including background metals and the other (group II) including metals that are considered potentially hazardous for the marine biota (Dadar et al. 2016; Mashroofeh et al. 2013). The inclusion of Fe in the second group could be related to localized anthropic sources, a phenomenon reported in the Iranian Caspian Sea also by Sohrabi et al. (2010) and confirmed by high iron concentrations in macroalgae. Hazardous elements, which are generally associated with human activities, followed a common increasing trend, while negative associations were observed with elements of the other group indicating their different origin in accordance with Bastami et al. (2014, 2017).

Notably, the highest concentrations of hazardous metals in sediments (corresponding to highly impacted conditions) were measured in the coastal tract affected by the discharge of Chalus River. In particular, this river is a potential source of Pb and Zn, due to the presence of the Sorb Dona mine in its upstream sector (Amini Rad et al. 2013; Jelodar et al. 2012), and data suggest that the upstream industrial activity produced a localized “footprint” clearly detectable in the coastal marine ecosystem. Pb and Zn can be transported downstream after absorption by sediments or suspended particles, mainly due to flood events (Amini Rad et al. 2013; Jelodar et al. 2012). Fast particle sedimentation rate in the coastal area may explain the detection of high concentrations of these metals only in the sites most directly affected by the river discharge. On the contrary, Ni and Cr exceeded their “non-pollution” levels at all sampling sites, confirming a large area of pollution. These two elements are commonly used as anti-fouling agents in marine paints (Tabari et al. 2010), and they can be also related to oil extraction (Alahverdi and Savabieasfahani 2012; Naser 2013). High concentrations of Ni were found in Anzali, where there is the most important port in the southern Caspian Sea, and in the two most touristic sites (Hachirud and Radio Darya), but moderate concentrations were found also in the other two sites. High concentrations of Cr were found in all sampling sites. These results suggest that the intense anthropic activity taking place at sea exposes the coastal waters to high levels of metal pollution even in less man-impacted site such as the area facing the Sisangan National Park.

Metals in sediments included elements able to spread along food chains up to fish consumed by man (Agah et al. 2007; Mashroofeh et al. 2013). Indeed, in the southern Caspian Sea, metals, in particular Hg, were found in the Persian sturgeon *Acipenser persicus* (Hosseini et al. 2013), and Cd, Pb, and Zn were found in inedible tissues of this and other two sturgeon species (Mashroofeh et al. 2013). Both studies concluded that the consumption of these fish species is safe, but both recommended further studies and monitoring programs.

As regards metals in algae, marked variations across sites and algal species were observed. Their concentrations were lower than in sediments for most of the elements. The macroalga *Enteromorpha* was widely distributed in the study areas compared to other algae that were only occasionally found (*Spirogyra* and *Sargassum*) and displayed the highest concentration for the majority of metals, thus representing an effective bioassessment tool in the investigated area. Some metal concentrations found in *Enteromorpha* can be considered indicative of impacted conditions according to literature (Fe, Caliceti et al. 2002; Zn, Villares et al. 2001; Cu, Chakraborty et al. 2014). Together, high concentrations of Fe, Cu, and Zn are usually associated to oil drilling, while Cu inputs can be associated also to antifouling products (Secrieru and Secrieru 2002; Tabari et al. 2010; Naser 2013).

No significant linear correlations were found between metal levels in algae and sediments. This result was expected, as macroalgae absorb metals from water (Bonanno and Orlando-Bonaca 2018), while sediments represent the long-term repository in aquatic environments. The absence of correlation also indicates a divergent partitioning of metals from water to these two basal compartments of the food web and possibly implies different effects of the herbivore and detrital energy pathways on the transfer of metals to upper trophic levels (Mendoza-Carranza et al. 2016; Signa et al. 2019).

## Conclusions

In summary, our results highlight (i) the ability of the macroalgal δ^15^N to identify the agricultural- and urban-derived N inputs in a closed water body affected by multiple, and otherwise not easy to identify, N sources, (ii) the general increasing trend in metal pollution, and (iii) the need to consider both macroalgae and sediments to obtain a reliable representation of metal pollution. As regards the first point, the macroalgal δ^15^N detected the origin of the N inputs at any DIN concentration in water. δ^15^N values indicated the signature of N inputs from synthetic fertilizers carried by the Sefid-Rud River into the coastal area of Anzali. In the central sites of the Chalus area, the macroalgal δ^15^N indicated N inputs mainly derived from tourism and municipal wastewaters.

The observed spatial variability of the macroalgal δ^15^N signatures and metal concentrations in sediments and macroalgae, as well as the lack of correlation between these parameters, indicates that the southern Caspian Sea is affected by multiple and independent anthropic inputs. Metal pollution derived from human activities taking place both in the inland and on the coast. Specifically, Cr and Ni were above the non-polluted thresholds in the sediments of all sampling sites, probably due to nautical and oil extraction activities. By contrast, Pb and Zn were well localized, affecting the touristic sampling sites, and were likely related to the Chalus River transportation from the Sorb Dona mine located upstream. Finally, the analysis of metals concentrations in macroalgae showed diffused high concentrations of Fe, suggesting the important role of oil extraction, mining, and industrial activities in the metal pollution.

In order to deepen the knowledge of the source, diffusion, and effects of pollution in the Caspian Sea, future studies on this complex ecosystem should explicitly consider the spatial variability of anthropogenic pressures and the land use cover. Furthermore, as already observed in the Caspian Sea (Agah et al. 2007) and in other coastal ecosystems (Signa et al. 2019), the potential harmful effects of metal pollution on higher trophic levels should be further investigated. Therefore, for a highly anthropized ecosystem such as the Iranian Caspian Sea, determining the effects of metal and nutrient pollution on the food web is necessary for effective management and conservation actions.

## Data Availability

The datasets used and/or analyzed during the current study are available from the corresponding author on reasonable request.
